# Secondary attack rates in primary and secondary school bubbles following a confirmed case: Active, prospective national surveillance, November to December 2020, England

**DOI:** 10.1371/journal.pone.0262515

**Published:** 2022-02-16

**Authors:** Annabel A. Powell, Georgina Ireland, Frances Baawuah, Joanne Beckmann, Ifeanyichukwu O. Okike, Shazaad Ahmad, Joanna Garstang, Andrew J. Brent, Bernadette Brent, Felicity Aiano, James Hargreaves, Sinéad M. Langan, Punam Mangtani, Patrick Nguipdop-Djomo, Joanna Sturgess, William Oswald, Katherine Halliday, Emma Rourke, Fiona Dawe, Zahin Amin-Chowdhury, Meaghan Kall, Maria Zambon, John Poh, Samreen Ijaz, Angie Lackenby, Joanna Elli, Kevin E. Brown, Sir Ian Diamond, Mary E. Ramsay, Shamez N. Ladhani

**Affiliations:** 1 UK Health Security Agency, London, United Kingdom; 2 East London NHS Foundation Trust, London, United Kingdom; 3 University Hospitals of Derby and Burton NHS Foundation Trust, Derby, United Kingdom; 4 Manchester University NHS Foundation Trust, Manchester, United Kingdom; 5 Birmingham Community Healthcare NHS Trust, Aston, United Kingdom; 6 Nuffield Department of Medicine, Oxford University Hospitals NHS Foundation Trust, Oxford, United Kingdom; 7 University of Oxford, Oxford, United Kingdom; 8 Faculty of Public Health and Policy, London School of Hygiene and Tropical Medicine, London, United Kingdom; 9 Faculty of Epidemiology and Population Health, London School of Hygiene and Tropical Medicine, London, United Kingdom; 10 Faculty of Infectious and Tropical Diseases, London School of Hygiene and Tropical Medicine, London, United Kingdom; 11 Office for National Statistics, Newport, United Kingdom; 12 Paediatric Infectious Diseases Research Group, St. George’s University of London, London, United Kingdom; Waseda University: Waseda Daigaku, JAPAN

## Abstract

**Background:**

Following the full re-opening of schools in England and emergence of the SARS-CoV-2 Alpha variant, we investigated the risk of SARS-CoV-2 infection in students and staff who were contacts of a confirmed case in a school bubble (school groupings with limited interactions), along with their household members.

**Methods:**

Primary and secondary school bubbles were recruited into sKIDsBUBBLE after being sent home to self-isolate following a confirmed case of COVID-19 in the bubble. Bubble participants and their household members were sent home-testing kits comprising nasal swabs for RT-PCR testing and whole genome sequencing, and oral fluid swabs for SARS-CoV-2 antibodies.

**Results:**

During November-December 2020, 14 bubbles were recruited from 7 schools, including 269 bubble contacts (248 students, 21 staff) and 823 household contacts (524 adults, 299 children). The secondary attack rate was 10.0% (6/60) in primary and 3.9% (4/102) in secondary school students, compared to 6.3% (1/16) and 0% (0/1) among staff, respectively. The incidence rate for household contacts of primary school students was 6.6% (12/183) and 3.7% (1/27) for household contacts of primary school staff. In secondary schools, this was 3.5% (11/317) and 0% (0/1), respectively. Household contacts were more likely to test positive if their bubble contact tested positive although there were new infections among household contacts of uninfected bubble contacts.

**Interpretation:**

Compared to other institutional settings, the overall risk of secondary infection in school bubbles and their household contacts was low. Our findings are important for developing evidence-based infection prevention guidelines for educational settings.

## Introduction

Early in the pandemic, the role of children in infection and transmission of SARS-CoV-2 was unclear and, therefore, many countries closed their educational settings as part of national lockdown to control the spread of the virus [1]. School closures not only affect the education of children but also have a profound effect on their mental, physical and social wellbeing, as well as access to social care, free school meals and school-based immunisations, all more likely to disproportionately affect the most disadvantaged and vulnerable families [2]. Despite these well-recognised consequences, parents and staff remain concerned about the risk of COVID-19 in educational settings, both to themselves and their household members.

In England, a rapid increase in SARS-CoV-2 infection during March 2020 led to school closures on 20 March 2020 followed by a nationwide lockdown on 23 March 2020 [3]. Cases increased until mid-April 2020 before declining until the end of May 2020 [4]. From 01 June 2020, some school years partially re-opened, with strict infection controls, including physical distancing, hand sanitisation and small class sizes or ‘bubbles’ of no more than 15 students [5]. Bubbles were created to separate children and staff into distinct groups with limited interactions with other bubbles, allowing rapid isolation of the bubble following a confirmed case, whilst allowing the remaining students to attend school safely. The definition of a bubble varied between schools, with most primary schools classifying individual class groups as single bubbles, whilst most secondary schools considered whole year groups as a bubble to allow pupils to mix across classes in the same year groups so that they have access to the whole curriculum of specialist subjects. Cases and outbreaks remained low during the six weeks of the summer half-term [5], and, along with similar successful experiences in other countries [6], this led to full reopening of all school years in England from September 2020.

Nationally, SARS-CoV-2 infections started increasing from mid-August 2020 (before schools re-opened), first in adults and then in children, leading to a tiered system of regional restrictions in October 2020, followed by national lockdown on 05 November 2020, although schools remained fully open [7]. Cases declined temporarily, again first in adults and then in children, but from 23 November 2020, following the emergence of the more transmissible Alpha variant (VOC 202012/01; B.1.1.7), cases rose rapidly across all age-groups in London and the South East before spreading across the rest of the country [7].

The large numbers of students returning to in-person schools from September 2020 posed extraordinary challenges for educational staff in implementing infection control measures. In particular, because of full-reopening, government advice issued in July 2020 recommended that the size of school bubbles was extended to include whole classes or year groups in instances where class-size bubbles were no longer possible, such as preventing students from accessing the full range of subjects, this was to be managed at the school ls discretion [8]. Consequently, the increasing number of cases in school-aged children between September and December 2020 led to large numbers of children and staff being sent home from school to self-isolate, often repeatedly, throughout the term [9]. The risk of SARS-CoV-2 infection in such bubbles, however, is not known and has been difficult to quantify. Emerging studies continue to demonstrate similar or lower risks of SARS-CoV-2 infection in schools compared to the local community [10–12], and active case-finding investigations report very few secondary transmission events in school premises following a confirmed case [13–15]. However, the high rate of asymptomatic infections, especially in students but also among educational staff [10, 11], raises the potential for silent transmission both within school and in households, which would not be identified through symptomatic testing only.

To assess the risk of SARS-CoV-2 infection in school bubbles, Public Health England (PHE) initiated the sKIDsBUBBLE study to test contacts of confirmed cases in school and their household members for SARS-CoV-2 infection and antibodies up to 30 days after bubbles were sent home to isolate. Measurement of SARS-CoV-2 antibodies provides a robust measure of SARS-CoV-2 exposure, capturing both symptomatic and asymptomatic infections. Whole genome sequencing of RT-PCR positive samples was also undertaken to identify the SARS-CoV-2 variants responsible for infection and to distinguish potential transmission chains from separate introductions of the virus within bubbles. Here we report the first results of school bubbles investigated at the end of the autumn term (17^th^ November– 15^th^ December 2020) in England.

## Methods

### Identification of a positive case

Laboratory-confirmed SARS-CoV-2 positive cases were identified in schools participating in the PHE COVID-19 Surveillance in School KIDs (sKIDs) (primary schools), sKIDsPLUS (secondary schools) or the Schools Infection Survey (SIS) (primary and secondary schools) [10, 11, 16]. Following a report of a SARS-CoV-2 case resulting in isolation of a bubble, PHE collected information as part of its public health investigation about the confirmed case from the school headteacher, including symptoms, test date and school attendance dates to assess the risk of exposure of the bubble contacts. The number of students and staff self-isolating as part of the bubble, was also collected, as well as symptoms and confirmed infections among the contacts. Only bubbles where the index case had been in school over the two school days before symptom start date or date of positive test were included in line with government advice on isolation of contacts [17].

### Recruitment of bubbles

Following agreement with the school headteacher, staff and parents of students (participants) in the self-isolating bubble were emailed by the school with information about the investigation and an online link to register, consent and complete a questionnaire via SnapSurvey. In addition to demographic data, the online questionnaire requested information about potential contact with any COVID-19 case and, if present, the start date and duration of any COVID-19 symptoms. Participants were also asked obtain consent from other household members and provide information about their demographics, and if present, symptoms. Participants completed an online questionnaire at enrolment and then at 7 and 30 days after enrolment.

### Testing

Participants were considered recruited once they provided online consent and completed the online questionnaire. Following recruitment, all household members received two nasal swabs for RT-PCR testing on Day 0 (on receipt of the kit) and Day 7, along with two oral fluid swabs for antibody testing on Day 0 and Day 30, with detailed instructions on self-sampling. Participants were instructed to post the samples to the PHE Colindale National Virus Reference Department on the same day the samples were taken. Nasal swabbing has similar sensitivity for detecting SARS-CoV-2 RNA as nasopharyngeal and oropharyngeal swabs but is a less invasive procedure [18]. These were tested by an RT-PCR assay on an Applied Biosystems 7500 FAST system targeting a conserved region of the open reading frame and envelope genes of SARS CoV-2 [19]. A positive RT-PCR result in a bubble participant was reported to the participant and headteacher, typically within 48 hours, and all household members were instructed to isolate as per national guidance at the time. Positive RT-PCR results in household members were reported directly to the participant. RT-PCR positive samples were sequenced by the Central Sequencing Laboratory in PHE Colindale. Oral fluid (OF) swabs tested for antibodies against the SARS-CoV-2 Nucleoprotein using an Immunoglobulin G capture based enzyme immunoassay [20].

### Statistical analysis

Data were managed in R Studio (v) and MS Access and analysed in Stata SE (version 15). Direct contacts are defined as the staff and students who were asked to self-isolate, indirect contacts refer to household members of these staff and students. For analysis, indirect contacts were separated into families where the bubble contact in the household subsequently tested positive for SARS-CoV-2 by RT-PCR or antibodies, compared to families where the bubble contact remained negative throughout the investigation. Denominators refer to the number of samples returned as not all those who returned the first samples returned the second nasal or oral fluid sample.

### Ethics approval

This investigation was undertaken as part of PHE’s duty, provided by Regulation 3 of The Health Service (Control of Patient Information) Regulations 2002 to (a) diagnose communicable diseases and other risks to public health; (b) recognise trends in such diseases and risks; (c) control and prevent the spread of such diseases and risks; and (d) monitor and manage outbreaks of communicable disease and incidents of exposure to communicable disease. The Health Service (Control of Patient Information) Regulations 2002 (legislation.gov.uk).

## Results

Between 17 November and 15 December 2020, 14 bubbles were recruited from seven schools, including 11 bubbles in four primary schools and three bubbles in three secondary schools (**Table 1**). All bar one of the primary schools defined individual classes as single bubbles, while one secondary school isolated only a single class and the other two isolated a whole year group. The median recruitment rate of self-isolating bubbles was 34.2% (269/786). Within the 14 bubbles, 269 direct contacts (248 students, 21 staff who formed part of the bubble) and 823 indirect contacts (524 adults, 299 children aged <18 years who were household contacts of the bubble contact) were enrolled and returned at least one sample to PHE. Of the direct contacts, 166/248 (66.9%) students and 17/21 (81.0%) staff returned both oral fluid samples. A student was the index case in 78.6% (11/14) bubbles.

**Table 1:** Demographics of participating direct contacts by bubble.

Bubble	Month	Year Group	Index Case	Students:Staff	F:M*	Bubble Total*	Participating n (%)
A	Nov	10	Student	49:0	16:33	188	49 (26.1)
B:1	Nov	2	Staff	5:4	6:3	35	9 (25.7)
B:2	Nov	3	Staff	3:0	1:2	30	3 (10.0)
C:1	Nov	R	Student	13:4	8:9	35	17 (48.6)
C:2	Nov	2	Student	14:3	7:10	35	17 (48.6)
D	Dec	5	Staff	18:0	9:9	77	18 (23.4)
E	Dec	12	Student	13:2	9:6	29	15 (51.7)
F	Dec	7	Student	84:0	44:40	191	84 (43.9)
G:1	Dec	3	Student	8:2	5:5	29	10 (34.5)
G:2	Dec	4	Student	6:2	6:2	27	8 (29.6)
G:3	Dec	4	Student	6:2	3:5	27	8 (29.6)
G:4	Dec	5	Student	9:1	6:4	31	10 (32.3)
G:5	Dec	5	Student	9:1	6:4	29	10 (34.5)
G:6	Dec	6	Student	11:0	5:6	23	11 (47.8)
Primary				102:19	62:59	378	121 (32.0)
Secondary				146:2	69:79	408	148 (34.8%)
Total				248:21	131:138	786	269 (34.2%)

*includes students and staff.

### Bubble contacts (direct contacts)

#### PCR testing

The median time between the bubble being sent home and receipt of the first sample at PHE was 9 (IQR, 7–11) days for sample 1 and 18 (IQR, 16–19) for sample 2. Overall, 2.9% (3/102) of primary school students were RT-PCR positive on sample 1, 2.1% (3/146) of secondary school students, 5.3% (1/19) of primary school staff and one of two (50.0%) secondary school staff. For sample 2, excluding those who were positive on sample 1, these numbers were 0% (0/70), 0.8% (1/119), 0% (0/16) and 0% (0/1) respectively (**Table 2**).

**Table 2:** RT-PCR and sequencing results of student and staff contacts by bubble.

Bubble	Students: Sample 1	Students: Sample 2	Staff: Sample 1	Staff: Sample 2	Index Sequence	Contacts Sequence
A	0/49 (0.0%)	0/46 (0.0%)	-	-	-	
B:1	0/5 (0.0%)	0/2 (0.0%)	1/4 (25.0%)	0/1 (0.0%)	Unknown	B.1.177
B:2	0/3 (0.0%)	-	-	-	-	
C:1	0/13 (0.0%)	0/12 (0.0%)	0/4 (0.0%)	0/4 (0.0%)	-	
C:2	0/14 (0.0%)*	0/13 (0.0%)	0/3 (0.0%)	0/3 (0.0%)	-	
D	2/18 (11.1%)	0/8 (0.0%)	-	-	Unknown	B.1.258 x 2
E	2/13 (15.4%)	0/8 (0.0%)	1/2 (50.0%)	0/1 (0.0%)	B.1.177.9	B.1.177.9 x 2, B.1.1.177.6 x 1
F	1/84 (1.2%)	1/65 (1.5%)	0/2 (0.0%)		B.1.177	B.1.1.7 x 2
G:1	0/8 (0.0%)	0/6 (0.0%)	0/2 (0.0%)	0/2 (0.0%)	-	
G:2	1/6 (16.7%)	0/5 (0.0%)	0/2 (0.0%)	0/2 (0.0%)	Unknown	B.1
G:3	0/6 (0.0%)	0/4 (0.0%)	0/2 (0.0%)	0/2 (0.0%)	-	
G:4	0/9 (0.0%)	0/8 (0.0%)	0/1 (0.0%)	0/1 (0.0%)	-	
G:5	0/9 (0.0%)	0/7 (0.0%)	0/1 (0.0%)	0/1 (0.0%)	-	
G:6	0/11 (0.0%)	0/5 (0.0%)	-	-	-	
Primary	3/102 (2.9%)	0/70 (0.0%)	1/19 (5.3%)	0/16 (0.0%)		
Secondary	3/146 (2.1%)	1/119 (0.8%)	1/2 (50.0%)	0/1 (0.0%)		
Total	6/248 (2.4%)	1/189 (0.5%)	2/21 (9.5%)	0/17 (0.0%)		

* one sample gave an indeterminable result.

#### Antibody conversion

Overall, 10.0% (6/60) of primary school students seroconverted between their first and second oral fluid sample, which was received at a median of 38 (IQR, 35–42) days after the start of isolation, 6.6% (7/106) of secondary school students, 6.3% (1/16) of primary school staff and no secondary school staff (**Table 3**). Those who seroconverted included five primary school students and four secondary school students who tested RT-PCR negative. None of the staff members seroconverted after having tested RT-PCR negative.

**Table 3:** Seroconversion rates of students and staff by bubble for all participants returning two oral fluid samples, and in those who were RT-PCR negative.

	STUDENTS		STAFF	
Bubble	All participants	RT-PCR negative participants	All participants	RT-PCR negative participants
A	0/41 (0.0%)	0/41 (0.0%)	-	-
B:1	0/2 (0.0%)	0/2 (0.0%)	1/3 (33.3%)	0/2 (0.0%)
B:2	0/1 (0.0%)	0/1 (0.0%)	-	-
C:1	2/10 (20.0%)	2/10 (20.0%)	0/3 (0.0%)	0/3 (0.0%)
C:2	1/10 (10.0%)	1/10 (10.0%)	0/3 (0.0%)	0/3 (0.0%)
D	1/4 (25.0%)	1/4 (25.0%)	-	-
E	3/7 (42.9%)	2/5 (40.0%)	0/1 (0.0%)	0/1 (0.0%)
F	4/59 (6.8%)	2/57 (3.5%)	-	-
G:1	0/6 (0.0%)	0/6 (0.0%)	0/2 (0.0%)	0/2 (0.0%)
G:2	1/5 (0.0%)	0/4 (0.0%)	0/2 (0.0%)	0/2 (0.0%)
G:3	0/3 (0.0%)	0/3 (0.0%)	0/2 (0.0%)	0/2 (0.0%)
G:4	0/6 (0.0%)	0/6 (0.0%)	-	-
G:5	1/7 (14.3%)	1/7 (14.3%)	0/1 (0.0%)	0/1 (0.0%)
G:6	0/6 (0.0%)	0/6 (0.0%)	-	-
Primary	6/60 (10.0%)	5/59 (8.5%)	1/16 (6.3)	0/15 (0.0%)
Secondary	7/106 (6.6%)	4/104 (3.9%)	0/1 (0.0%)	0/1 (0.0%)
Total	13/166 (7.8%)	9/162 (5.6%)	1/17 (5.6%)	0/16 (0.0%)

#### Symptoms

Of the 16 children who were SARS-CoV-2 positive on RT-PCR and/or became antibody positive, three had typical symptoms, including cough, fever and/or anosmia, of SARS-CoV-2 infection, five reported atypical symptoms such as sore throat, runny nose, abdominal pain, headache or fatigue and eight were asymptomatic.

#### Whole genome sequencing

Of the 14 bubbles, a direct contact was RT-PCR positive in 5 (35.7%) bubbles; 3/11 primary and 2/3 secondary school bubbles. Whole genome sequencing was successful in two of five index cases (the initial confirmed case that led to the bubble self-isolating) and all nine positive direct contacts (**Table 2**). Where the sequence was known for the index case and their direct contact in the bubble, 60% (3/5) were different to the index case and in-school transmission between the two could be discounted. Bubble E had 3 positive cases among direct contacts in addition to the index case; two were students and had the same strain as the index case (B.1.177.9) while one, a teacher, was different (B.1.177.6). Bubble F had two positive cases among direct contacts in addition to the index case, which were both different phylotypes of B.1.1.7 to each other and a different strain to the infecting the index case (B.1.177.6), indicating that transmission did not occur between any of these individuals. For Bubble D, the index case strain was unknown but the two cases among the direct contacts were due to the same strain, B.1.258. Overall, four of the nine sequences available for comparison identified different SARS-CoV-2 strains, therefore, ruling out transmission between affected individuals (**Table 2**).

### Households contacts (indirect contacts)

**Fig 1:**
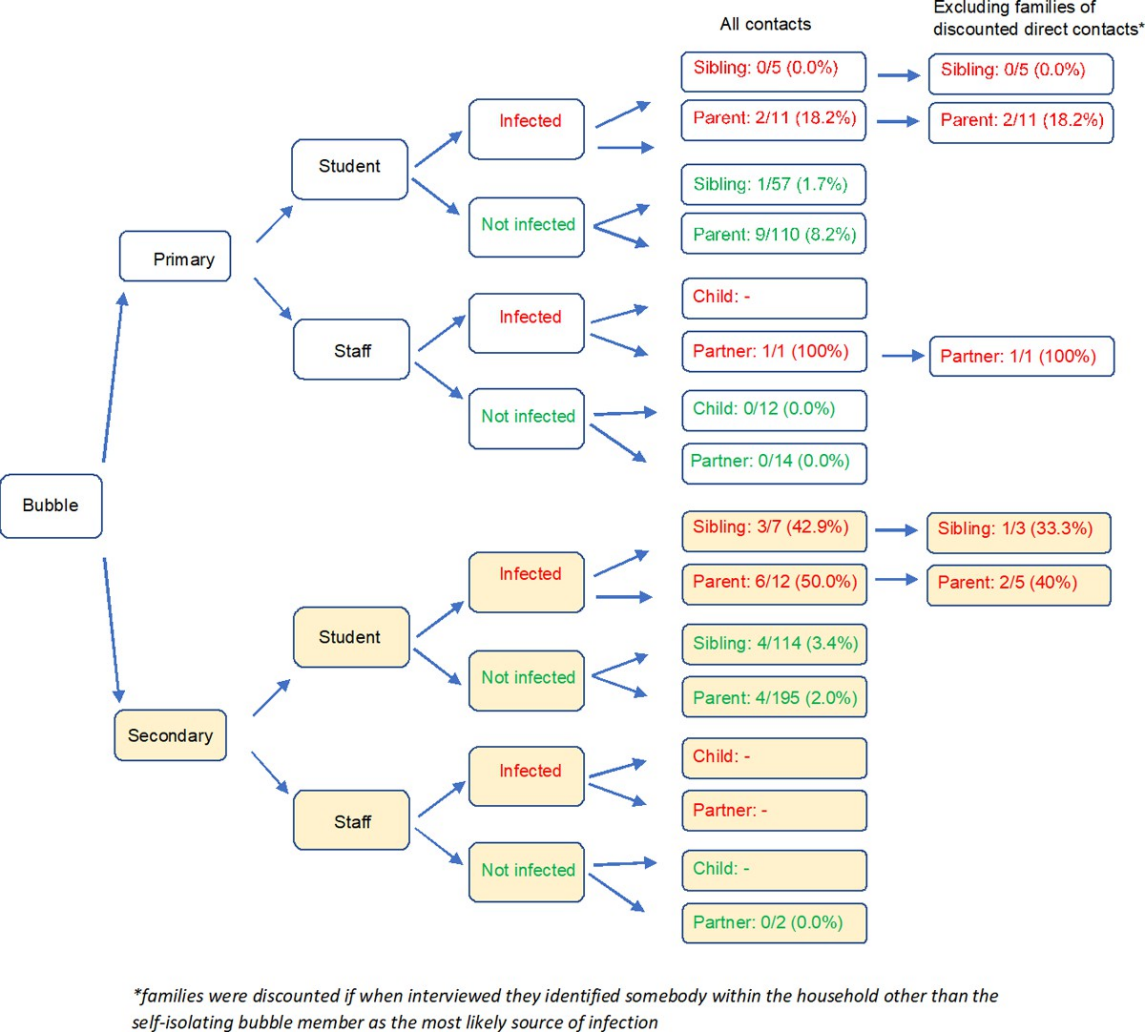
Flow diagram showing the secondary attack rates of indirect contacts.

In primary school households where the student contact was SARS-CoV-2 positive (RT-PCR positive or seroconverted), 33.3% (2/6) had an additional household member who also tested positive, including 18.2% (2/11) parents but no siblings (0/5) (**Fig 1**). When interviewed, both these households identified the most likely primary case within the family as the student attending school. The single staff member self-isolating as part of a primary school bubble and testing SARS-CoV-2 positive had one participating adult household member who also tested positive subsequently (1/1, 100%).

In secondary school households where the student was SARS-CoV-2 positive, 87.5% (7/8) had an additional household member also test positive, including 50% (6/12) parents (all RT-PCR positive) and 42.9% (3/7) siblings, of whom 2/3 had tested RT-PCR negative but seroconverted. When interviewed, however, four of the seven households with an additional household member who also tested positive said that the suspected primary case was not the bubble participant but another family member.

In households of students who were bubble contacts but had no evidence of infection (RT-PCR and antibody positive), we identified siblings and parents who had evidence of SARS-CoV-2 infection, more so in households of primary (10/167, 6.0%) than secondary (8/309, 2.6%) school students contacts. Staff contacts who had no evidence of infection did not have any household members with SARS-CoV-2 infection, although numbers tested were small (**Fig 1**).

#### Secondary attack rates

Overall SARS-CoV-2 infection rate among students was 10.0% (6/60) in primary and 6.7% (8/106) in secondary school bubbles. (**Table 4**) After analysis of sequencing data and interviewing participants, the secondary attack rate in primary school children was estimated to be the same but reduced to 3.8% (4/102) in secondary school students. Among staff, secondary attack rates were 6.3% (1/16) and 0.0% (0/1), respectively, but were small numbers.

**Table 4:** Secondary attack rates of students and staff including only those that returned one RT-PCR sample and both oral fluid samples.

Participant Type	No evidence of infection	Sample 1 PCR positive	Sample 2 PCR positive	Seroconverted*	Attack rates
a) Overall					
Median time to sample returned (IQR)		9 (7–11)	18 (15–19)	38 (35–42)	
Primary student	54/60 (90.0%)	1/60 (1.6%)	0/60 (0%)	5/60 (8.33%)	6/60 (10%)
Secondary student	98/106 (92.5%)	3/106 (2.8%)	1/104** (1.0%)	4/106 (3.8%)	8/106 (7.5%)
Primary staff	15/16 (93.8%)	1/16 (6.3%)	0/16 (0%)	0/16 (0%)	1/16 (6.3%)
Secondary staff	1/1 (0%)	0/1 (0.0%)	0/1 (0%)	0/1 (0%)	0/1 (0%)
Total	168/183 (91.8%)	5/183 (2.7%)	1/181 (0.6%)	9/183 (4.9%)	15/183 (8.2%)
b) Excluding cases who were discounted due to phylogenetics or interviews					
Median time to sample returned (IQR)		9 (7–11)	18 (15–19)	38 (35–42)	
Primary student	54/60 (90.0%)	1/60 (1.6%)	0/60 (0.0%)	5/60 (8.3%)	6/60 (10.0%)
Secondary student	98/102 (96.1%)	2/102 (2.0%)	0/100** (0.0%)	2/102 (2.0%)	4/102 (3.9%)
Primary staff	15/16 (93.8%)	1/16 (6.3%)	0/16 (0.0%)	0/16 (0.0%)	1/16 (6.3%)
Secondary staff	1/1 (100%)	0/1 (0.0%)	0/1 (0.0%)	0/1 (0.0%)	0/1 (0.0%)
Total	167/179 (93.3%)	4/179 (2.2%)	0/179 (0.0%)	7/179 (3.9%)	11/179 (6.1%)

*Includes only those who were RT-PCR negative.

Using all available evidence, the incidence rate of household contacts of primary school students isolating as part of a bubble was 6.6% (12/183) and 3.7% (1/27) for household contacts of self-isolating primary school staff. For household contacts of secondary school students, this was 3.5% (11/317) and none for (0/1) secondary school staff.

## Discussion

Active investigation of 14 school bubbles sent home for self-isolation following a single confirmed case of COVID-19 in the bubble identified additional cases in 7 bubbles. The secondary attack rate was 10.0% in primary and 3.8% in secondary school students and, although fewer staff members were involved, secondary attack rates were 6.3% and 0%, respectively. Swabbing of bubble contacts only would have identified fewer than half the cases in students, most of whom were asymptomatic throughout the surveillance period. Whole genome sequencing identified almost half the cases in individual bubbles to be due to different SARS-CoV-2 strains. Among households with a positive bubble contact, 2/6 primary and 7/8 secondary school students had another member who also tested positive during the self-isolation period although, in more than half the affected secondary school households, the family reported the source of infection to be someone other than the student bubble contact. We also found evidence of new infection among household members of bubble contacts who were not infected, more so among household members of primary than secondary school students.

Our estimates of secondary attack rates in students are higher than other school investigations, including those with active case finding [9, 13–15, 21], because we employed active and multiple testing to capture both asymptomatic, symptomatic and mild, transient infections over a 30-day period after self-isolation. Our findings also highlight the added value of antibody testing which identified more than twice as many infected students than PCR alone, similar to our school serosurveillance studies [10, 11]. This was particularly the case for students, who were less likely to be symptomatic or test RT-PCR positive, and more likely to seroconvert in the absence of symptoms or RT-PCR positivity, than staff [10, 11].

Our estimates of secondary attack rates are likely to be an overestimate, because of difficulties in ascertaining the source and direction of infection despite extensive testing, questionnaire completion and interviewing of families. This was evidenced by the high rates of asymptomatic infections where it was not possible to ascertain the timing of infection or infectious period, the identification of different strains within the same bubble, families reporting infection sources other than the bubble contact and confirmed infections in household members of student bubble contacts who were themselves not infected.

These attack rates, however, are substantially lower than other institutional settings, especially care homes (78%) [22], but also hospitals (36%) [23], prisons (58%) [24], and detention centres (50%) [25], despite children having higher contact patterns than adults, especially in school [26]. The overall risk to household contacts of isolating bubble members is also low although, if a bubble member becomes infected, then the risk to their household contacts is similar to rates reported in household SARS-CoV-2 transmission studies [27]. Our results are consistent with more recent outbreak investigations involving active case-finding, which found that, when adequate infection control measures are in place, most cases in school are due to multiple separate introductions rather than in-school transmission, which accounted for less than 5% of total cases [13–15].

In the current investigation, most secondary schools self-isolated as part of larger year groups while primary schools self-isolated in smaller classes, which likely reflects the higher secondary attack rates in the latter where children may be more likely to have close contact with the confirmed cases. A consequence of larger bubbles following the full reopening of all school years was that the ratio of children self-isolating for every case also increased. On 08 July 2021, for example, 11.2% of pupils attending state schools in England were not in class for COVID-19 related reasons, including 750,000 self-isolating due to potential contact with a COVID-19 case, compared to 35,000 self-isolating because of suspected COVID-19 and 39,000 with confirmed SARS-CoV-2 infection [28]. Many students have had to self-isolate multiple times during the academic year, including those with confirmed COVID-19, who have a very low risk of re-infection [29].

The extensive disruption to their education raises the question as to whether bubbles need to self-isolate following a single case in the bubble. On the one hand, the risk of infection among bubble contacts was higher than background population rates of 0.9% at the time [30], and at least some of the infections in bubble contacts were acquired following contact with the confirmed case in the bubble. This would suggest that the infection could spread through–and potentially across bubbles–if they are not isolated from school. Isolating infected bubbles has the potential to break chains of transmission allowing all the other students to remain in school. On the other hand, repeated self-isolation is highly disruptive to their education and, importantly, a recent clinical trial involving 200 schools across England found that daily contact testing of students contacts with lateral flow devices (LFD) whilst remaining in school was non-inferior to self-isolation for control of COVID-19 [31]. The finding of <2% SARS-CoV-2 infections in both intervention and control groups through PCR testing is consistent with our findings and we have further demonstrated an overall low risk of infection among household contacts of bubble contacts. Importantly, in the UK the COVID-19 vaccination programme began on the 8 December 2020 with all adults eligible for COVID-19 vaccination from 17 June 2021 and subsequently adolescents aged 16–17 from 4 August 2021 and those aged 12–15 from 13 September [32–35]. This will help protect eligible students, education staff and household members against severe disease if exposed to the virus as well as potentially reduce outbreaks in schools by interrupting transmission.

### Strengths and limitations

We captured asymptomatic and symptomatic infections using both RT-PCR and antibody testing as well as using whole genome sequencing to better understand transmission. Our findings provide a risk estimate of SARS-CoV-2 infection among bubble contacts and their household members but, for some events, we were unable to distinguish whether the infection was acquired from the confirmed case in the bubble or elsewhere. There are some limitations. Firstly, only a third of bubble contacts agreed to participate and, in some bubbles, the family of the index case declined to participate. We have no additional information apart from the bubble size to determine whether those agreeing to take part were representative of the whole bubble. Additionally, there were unavoidable delays in engaging schools and allowing families enough time to participate in the investigation. Consequently, the median time from self-isolation to the first sample was 9 days, which meant that we may have missed some co-infection at the same time as the index cases and the early secondary infections in the bubble. Additionally, the small number of participating staff limited interpretation of our findings in this population. We also have no information on the infecting strains in students who seroconverted but remained RT-PCR negative. The oral-fluid assay has many advantages over blood sampling for antibody testing since it can be performed by the participant in their homes. However, the estimated sensitivity is around 80% when compared to contemporaneous serum samples, which may therefore miss some seroconversions [20]. Our investigations were conducted mainly in London when the Alpha variant emerged and when adults were in national lockdown, which would affect transmission dynamics, especially within households, but this should reduce new introductions of the virus and, therefore, provide more accurate estimates of secondary attack rates. Finally, our estimates may not be applicable to other variants, including the Delta variant, which is currently responsible for nearly all SARS-CoV-2 infections in England.

## Conclusions

We have estimated the secondary attack rates following a confirmed case in school bubbles and their household contacts during a period of moderate-to-high community transmission with a more transmissible Alpha variant. With the availability of regular home-testing kits for education staff and students and the option for daily home-testing in exposed individuals, along with high vaccination rates in adults and adolescents, every effort should be made to reduce the need for repeated isolations and disruption to children’s education.
